# PP68 Successfully Streamlining New Medicines Assessments In Scotland: A Stakeholder Evaluation Of The Scottish Medicines Consortium’s Abbreviated Submission Process

**DOI:** 10.1017/S0266462324002393

**Published:** 2025-01-07

**Authors:** Alex Henriquez, Pauline McGuire, Christine Hepburn

## Abstract

**Introduction:**

The Scottish Medicines Consortium (SMC) assesses new medicines for NHSScotland, based on company submissions. The abbreviated process previously applied to new formulations of medicines with anticipated low net budget impact. During the COVID-19 pandemic, SMC extended the abbreviated process to new medicines with alternatives within the same therapeutic class already approved in Scotland. This evaluation explored stakeholder perspectives on this process

**Methods:**

A mixed-method approach, with focus groups and online surveys, was utilized to collect data from stakeholders (n=56) including Patient Group Partners (n=14), Public Partners (n=2), pharmacists employed in local health boards and in national procurement (n=17), pharmaceutical industry representatives (n=10), SMC senior clinical assessors (n=6), and the SMC Executive Team (n=7). All participants were asked a series of open and semi-open questions about specific aspects of the process, its advantages, disadvantages, and potential areas for improvement. A thematic analysis, based on initial color coding and grouping of responses into themes, was employed to analyze the qualitative data

**Results:**

Overall, the abbreviated process was commended for its time and resource efficiency, particularly benefiting the SMC assessment teams, decision-making committees, and the pharmaceutical industry. The decision-making committees experienced a decrease in workload and capacity gains, which allowed them to focus on more innovative new medicines. Overall, stakeholders welcomed rapid access to new medicines, and resource savings for pharmaceutical companies, patient groups, and the SMC assessment teams. Suggestions for improvement included strengthening patient group involvement in the process, improving transparency of the decision-making process, in particular the budget impact assessment, and providing further guidance on the definitions of “same therapeutic class.”

**Conclusions:**

Regulatory agencies are licensing more new medicines, in terms of volume and complexity. HTA bodies must consider innovative ways to undertake proportionate assessments. Extension of SMC’s abbreviated process was well commended by stakeholders, primarily due to resource-savings benefits and expedited access to new medicines. Evaluation of the change identified further areas for improvement.

